# Cluster analysis identifies long COVID subtypes in Belgian patients

**DOI:** 10.1093/biomethods/bpae076

**Published:** 2024-10-09

**Authors:** Pamela Mfouth Kemajou, Tatiana Besse-Hammer, Claire Lebouc, Yves Coppieters

**Affiliations:** School of Public Health, Centre for Research in Epidemiology, Biostatistics and Clinical Research, Université Libre de Bruxelles (ULB), B-1070 Brussels, Belgium; Department of Internal Medicine, Faculty of Medicine, Universite Libre de Bruxelles (ULB), B-1070, Brussels, Belgium; Clinical Research Unit, Centre Hospitalier Universitaire Brugmann, 1020, Brussels, Belgium; Clinical Research Unit, Centre Hospitalier Universitaire Brugmann, 1020, Brussels, Belgium; School of Public Health, Centre for Research in Epidemiology, Biostatistics and Clinical Research, Université Libre de Bruxelles (ULB), B-1070 Brussels, Belgium

**Keywords:** SARS CoV-2, long COVID, phenotypes, clusters, clinical forms

## Abstract

Severe acute respiratory syndrome coronavirus infection presents complications known as long COVID, a multisystemic organ disease which allows multidimensional analysis. This study aims to uncover clusters of long COVID cases and establish their correlation with the clinical classification developed at the Clinical Research Unit of Brugmann University Hospital, Brussels. Such an endeavour is instrumental in customizing patient management strategies tailored to the unique needs of each distinct group. A two-stage multidimensional exploratory analysis was performed on a retrospective cohort of 205 long COVID patients, involving a factorial analysis of mixed data, and then hierarchical clustering post component analysis. The study's sample comprised 76% women, with an average age of 44.5 years. Three clinical forms were identified: long, persistent, and post-viral syndrome. Multidimensional analysis using demographic, clinical, and biological variables identified three clusters of patients. Biological data did not provide sufficient differentiation between clusters. This emphasizes the importance of identifying or classifying long COVID patients according to their predominant clinical syndrome. Long COVID phenotypes, as well as clinical forms, appear to be associated with distinct pathophysiological mechanisms or genetic predispositions. This underscores the need for further research.

## Introduction

Coronavirus disease 2019 (COVID-19), caused by the severe acute respiratory syndrome coronavirus, SARS-CoV-2, is an emerging infectious disease. Like SARS-CoV-1, its predecessor, which emerged in 2003 [1], SARS-CoV-2 infection can lead to persistent complications affecting various organ systems collectively referred to as Post-COVID Syndrome or PASC (Post-Acute Sequelae of SARS-CoV-2) or long Haulers COVID or long COVID [2, 3]. These complications impact approximately 10%–20% of COVID-19 patients, as defined by the World Health Organization (WHO), which characterizes long COVID as ‘ the continuation or development of new symptoms 3 months after the initial SARS-CoV-2 infection, with these symptoms lasting for at least 2 months with no other explanation’ [4].

Long COVID encompasses a spectrum of multisystemic alterations, with various pathophysiological mechanisms that overlap, impairing the development of a comprehensive treatment approach [5–7]. While numerous symptoms associated with long COVID have been described, none are specific to the disease, but all can be debilitating, especially when they persist for over a year [8].

Immunological and clinical forms of long COVID are well documented [9, 10], and statistical models, such as latent class analysis and machine learning, have identified clusters of patients with similar characteristics [11–13]. However, no consensual definition has been developed, likely explaining the multitude and diversity of phenotypes observed [10, 13, 14].

One study characterized long COVID 3 months after infection into three distinct phenotypes [14]. Kenny *et al*. also identified three phenotypes 4 weeks after infection, while Frontera *et al*. approximately 1 year after SARS CoV-2 infection, identified three phenotypes or clusters as well, but with different affected systems [15, 16].

The differences observed in the composition of clusters or phenotypes in various studies result from specific symptomatology associated with each virus strain, and therefore specific to each wave of infection, as suggested by an Italian study [17]. Additionally, they may result from changes in clusters across times which might be dynamic [18, 19].

WHO and NICE (National Institute for Health and Care Excellence) guidelines defined long COVID with various onset period, from 1 to 3 months [20, 21], also probably explaining the diversity of phenotypes. Based on symptoms time course, Davis *et al*. described three clusters: Cluster 1 consists of symptoms that are most likely to occur early in the illness, reaching a high point in the first 2 or 3 weeks, then decreasing in probability over time. Cluster 2 consists of symptoms with a relatively stable probability over time. Cluster 3 consists of symptoms most likely to increase sharply in the first 2 months [22]. Chen *et al*. identified three phenotypic clusters associated with long COVID time course that they characterized as remittent, persistent, or incident based on the 3-month change in symptom number compared to study entry [23]. Another study has identified seven different types of symptoms’ time course: (i) symptoms were very intense at first 3–4 weeks and progressively decrease; (ii) symptoms increase their intensity for the first 3–4 weeks and have not decrease its intensity; (iii) symptoms have maintained same intensity from the beginning since nowadays; (iv) symptoms were of high intensity for the first 3–4 weeks and then persist, intensifying, in a cyclical way, without disappearing completely; (v) symptoms were of high intensity for the first 3–4 weeks and after that, decreased their intensity fluctuating, until they disappear; (vi) symptoms intensity do not follow any pattern that I can identify; (vii) no graphic represents my perception of my symptom’s evolution over time [24].

An empirical classification based on distinct symptom onset, symptom timing course, biological and clinical characteristics has been devised at the Clinical Research Unit of Brugmann University Hospital, Belgium with three major syndromes recognized. This clinical classification includes:

- Hypoxemic long COVID patients who required oxygen therapy and often intensive care during acute COVID-19 illness, due to respiratory failure necessitating intubation. Their oxygen saturation levels were, at least on one occasion, less than 89% on ambient air and at rest. Nonetheless, some healthcare professionals equate this form with PICS (Post Intensive Care Syndrome) and the complications that arise from being hospitalized in an ICU (Intensive Care Unit) as they share physical, neurological, and psychiatric complaints and the distinction between the two entities is slight (pulmonary fibrosis and bronchiectasis are common in COVID-19 ICU-patients but not specific) [25, 26]. According to CDC, Post-COVID Conditions is a set of three conditions amongst long COVID, multiorgan effects of COVID-19 and effects of COVID-19 treatment and hospitalization: PICS might be a result of COVID-19 treatment and hospitalization [27].
- “Persistent forms” encompass patients who, following an initial episode of influenza-like illness, experienced multi-organ symptoms that worsened (with the sensation of multiple relapses) from 4 to 8 weeks after infection.
- The “long” forms encompass patients who, within an interval of 1–4 weeks, predominantly developed neurocognitive symptoms, followed by multi-organ symptoms, organ by organ. It appears that each “recurrence” led to new symptomatology and exacerbated existing symptoms.

In addition to these three syndromes, two other syndromes are recognized in the literature, namely mast cell activation syndrome (MCAS) and post-viral syndrome [28, 29]:

- The post-viral or “post-COVID syndrome” category represents individuals who do not fit into any of the three primary clinical forms aforementioned. Post viral syndrome is described as the consequence of viral infections such as EBV (Ebstein Barr virus), HSV (Herpes Simplex Virus) and it is not specific to any infection [30, 31].
- The MCAS, which primarily presents with digestive symptoms and is diagnosed through tryptase assays [32]. The clinical presentation of MCAS can also involve the cardiopulmonary, gastrointestinal, dermatological, and neurological systems. One study even suggests that MCAS during COVID-19 infection leads to Long COVID and it is also known that long COVID can trigger MCAS [29].

Beyond this clinical classification, various statistical methods including unsupervised machine learning and clustering have been used to categorize long COVID patients into clusters [15, 33, 34]. However, some of these studies did not consider biological criteria, and others overlooked common clinical signs, such as dysautonomia manifested by exercise intolerance, dyspnoea, and Postural Orthostatic Tachycardia Syndrome (POTS). In this study, we employ comprehensive clinical and biological criteria to identify distinct clusters of long COVID more precisely and hypothesize that there exist connections between clusters and the aforementioned empirical clinical forms.

## Methods

### Study design and participants

We conducted an exploratory analysis from January to May 2023 at Brugmann University Hospital, Brussels, Belgium from a retrospective study of medical reports where convenience sampling was used, more precisely purposeful sampling (participants were directly selected by the researcher). After the medical examination and anonymization of patient records, only the principal investigator could identify the study participants individually. Collected anonymized data were then coded for analysis purpose and to preserve confidentiality and security of the participants.

Medical records were provided from a cohort study initiated in May 2020 at Brugmann University Hospital to longitudinally monitor patients with a probable or confirmed COVID-19 diagnosis whose symptoms persisted and worsened for more than a month or who developed new symptomatology, even though tests did not reveal the presence of SARS CoV-2 infection or another condition.

### Inclusion and exclusion criteria

The study included individuals aged 18 or older who had a probable or confirmed diagnosis of COVID-19, and who had been clinically, radiologically, and neuropsychologically diagnosed with long COVID. Probable cases of COVID-19 were included from the initiation of the study due to the lack of diagnostic tests during the first wave of the infection. Furthermore, a study has identified two immunological types of long COVID, one of which presented with seronegative test following SARS CoV-2 infection [9]. The long COVID condition was suspected clinically and confirmed later with biological, radiological, and neuropsychological tests. Participation in the study was contingent upon the individuals' voluntary and informed consent. Individuals who had been previously diagnosed with Alzheimer's disease, demyelinating pathology, dementia, or mild cognitive impairment, patients with cancer, pregnant women or those with 70% missing information in their records, were excluded from the study.

### Ethics

This study was subject to prior approval by the Ethics Committee of the Brugmann University Hospital (ref CE 2022/56) to comply with the principles of the Declaration of Helsinki. Informed consent was required from participants prior to inclusion in the study and before collecting data. The data collected were coded to preserve data confidentiality and patient safety.

### Clinical assessments

Upon obtaining their voluntary consent, participants underwent a comprehensive medical history collection, and a meticulous clinical examination was conducted to identify clinical signs of long COVID, particularly dysautonomia and psychiatric disorders (anxiety and/or depression).

For patients without psychiatric disorders, the following assessments were conducted: Nijmegen tests to diagnose respiratory hyperventilation [35, 36], the 5-word test to diagnose cortico-subcortical pathology [37, 38], the MacNair Cognitive Difficulties Self-Rating Scale (CDS) to screen for neurocognitive disorders [39], a specific MacNair test to evaluate memory [40].

Symptoms were documented in chronological order, along with their duration, during the inclusion phase, and at 6 and 12 months thereafter.

### Biological assessments

Data pertaining to various biological tests were also collected, which were performed in the post-COVID period and brought in by the patients. For patients with clinically suspicious MCAS, a tryptase assay was conducted. When there was not a confirmed diagnosis of acute SARS CoV-2 infection, due to unavailability or negativity of tests during suspicious acute phase (probable infection), CRP, IL-6 and one serological test were indicated respectively to rule out new infection, to confirm long COVID syndrome and to corroborate ancient SARS CoV-2 infection [41, 42]. If necessary, additional samples were collected to further assess endothelial dysfunction later.

### Radiological and neuropsychological assessments

Following the clinical and biological assessments, neurocognitive testing was conducted to corroborate the patients' subjective complaints. If neurocognitive testing yielded any deficit such as attention or memory impairment, patients underwent brain scintigraphy followed by neurocognitive rehabilitation. In cases where neurocognitive testing yielded no impairments, patients were recontacted 1 year later to monitor changes in their symptoms. Patients without cognitive complaints did not perform scintigraphy. When neurocognitive test yielded negative results, neurorehabilitation was not performed.

### Patients’ management

For patients with psychiatric disorders, specifically anxiety and/or depression, a general questionnaire was administered to determine whether the COVID period had induced any stress. The following tests were then carried out: Hospital Anxiety and Depression Scale for assessing anxiety and depression [43], PCL-S (Post-traumatic Checklist Scale) to diagnose Post-traumatic Stress Disorder [44], Cognitive Failure Questionnaire for diagnosing cognitive disorders [45, 46], Eight-item Somatic Symptom Scale for diagnosing complaints related to fatigue [47].

If any of these four scales indicated abnormalities, patients were referred for psychiatric consultation and treatment. If neurocognitive remediation was deemed necessary, neurocognitive testing was conducted prior to the treatment. Subjective complaints were longitudinally monitored at 6, 12, and 24 months. If none of these scales indicated abnormalities, patients were reevaluated after 22–26 weeks, and the screening scales were repeated.

Psychiatric referrals to various clinics were made based on the following scales: State Trait Anxiety Inventory form Y-B for anxiety traits [48], Beck Depression Inventory for depression [49], Impact of Event Scale to measure stress perception related to a traumatic event [50], Childhood Trauma Questionnaire [51], Resilience Scale, SF-36 (Short-Form 36) quality of life Scale [52, 53], Montreal Cognitive Assessment [54], PCL-S, Fatigue Severity Scale [55], Visual Analog Scale for pain assessment [56], neurological Pain Assessment Scale [57], Body chart, Modified Borg scale to assess the perceived intensity of effort during exercise (e.g., a 6-min walking test) [58].

### Variables

The variables described were represented by:

- Demographic variables: age, gender, clinical form of PASC and wave included.
- Variables listing medical history: obesity, allergy, smoking, autoimmune disease, ischaemic heart disease, hypertension, chronic obstructive pulmonary disease.
- Clinical variables: concentration disorders, memory disorders, fatigue, phasic disorders, sleep disorders, anxio-depressive syndrome, dyspnoea, headaches, dysautonomia, mood disorders, myalgia, joint pain, paraesthesia, digestive disorders, chest pain, palpitations, visual disturbances, exertional intolerance, auditory disturbances, dizziness, taste disturbances, olfactory disturbances, muscle weakness, alopecia, abnormal movements, cough, alcohol intolerance, weight loss.
- Biological variables: c-reactive protein, lacticodehydrogenase, d-dimer, creatine kinase, alanine aminotransferase, aspartate aminotransferase, neutrophils, lymphocytes, platelets, blood calcium, creatininaemia, vitamin D, fasting glycaemia, total cholesterol, high density lipoprotein cholesterol, LDL (low density lipoprotein) cholesterol, alkaline phosphatase, ferritinaemia.
- Time variables: time between onset of COVID-19 symptoms and inclusion in the study, time between infection and assessment, time between onset of infection and long COVID, time between onset of long COVID and inclusion in the study.

Only demographic, clinical, and biological variables were included in multidimensional analysis in order to identify patterns of long COVID. Biological variables such as creatine kinase, alkaline phosphatase, d-dimer, and vitamin D were not included in multidimensional analysis due to important missing values.

### General statistical analysis

Descriptive analyses were conducted to assess the distribution of various variables. Normally distributed continuous variables were summarized using the mean and standard deviation, while non-normally distributed variables were summarized using the median and interquartile range (IQR). Categorical variables were presented as frequencies. A bivariate analysis was performed to identify variables that were correlated with each other. The Cramer's V test was used to examine the relationship between two qualitative variables, while Pearson's linear correlation coefficient was used to investigate the relationship between two quantitative variables. If a variable exhibited a high correlation with another, one of them was excluded from the multivariate analysis.

### Multidimensional analysis

An exploratory analytical approach utilized two complementary statistical techniques known as multidimensional data analysis methods. These techniques facilitated the creation of synthetic representations of objects (such as individuals, variables, and modalities of qualitative variables) in the form of point clouds within a Euclidean space, which could be reduced to a two-dimensional plane. The Euclidean aspect meant that distances between points and angles for quantitative variables were interpreted in terms of correlation for quantitative variables and similarity for individuals and modalities.

### Factor analysis of mixed data

Factorial analysis of mixed data (FAMD) is a principal component analysis method that combines the transformation of categorical data into coordinates in a multidimensional space, with quantitative data transformation. It aims to assess linear relationships between variables by detecting the main dimensions of variability and summarizing them into synthetic variables, thereby reducing the number of variables needed to represent the data.

Before conducting FAMD, several steps were taken:

- Bivariate analysis was performed to exclude highly correlated variables. For instance, LDL cholesterol and total cholesterol levels were highly correlated, leading to the exclusion of the total cholesterol variable from the analysis.
- Active variables (clinical and biological) were distinguished from additional variables that did not contribute to calculating distances between individuals. These additional variables were age categories, gender, wave of SARS-CoV-2 infection, and long COVID clinical form. They were considered explanatory variables but did not participate in constructing the axes.
- A balanced distribution of modalities was ensured to avoid bias in the factorial analysis. Variables with a frequency of less than 10% were excluded from the active variables since they could not be further subdivided into multiple modalities.
- Missing data were imputed by estimating the value of variable *x* from that of *y* if they were correlated, or by estimating the value of *x* from an individual with similarities to it.

During the factorial analysis, an outlier was detected, which had a disproportionate influence on constructing the axes and was subsequently removed from the analysis [59].

### Cluster analysis or HCPC

Secondly, cluster analysis or HCPC was used on the coordinates of the mixed analysis using aggregation by inertia to obtain clusters or a partition of the data. This method starts with each participant as its own group, combining the most “similar” participants based on proximity of Euclidean distance, continuing until the last groups merge into 1 group containing all participants: this is Ward's method. The clusters were then consolidated to make them homogeneous. The clusters were compared using the *χ*^2^ test for categorical variables and the Kruskal–Wallis test for continuous variables.

## Results

### Study participants

**Figure 1.**
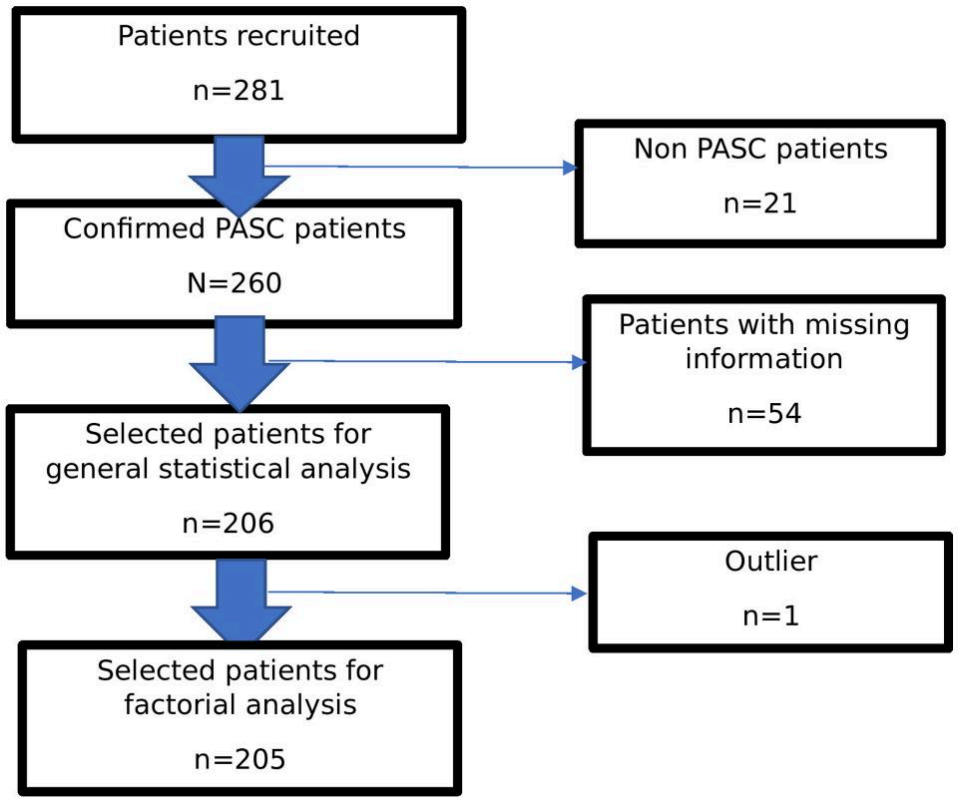
Patient file selection flow chart.

A total of 281 patients were recruited until May 2023. Twenty-one were excluded due to having conditions other than PASC which could interfere with neuropsychologic tests. Of the remaining 260, 206 were included in this study, while 54 were excluded due to insufficient data. Additionally, an outlier was identified during factorial analysis and subsequently excluded (Fig. 1).

Participants were recruited, on average, 328 ± 176 days (approximately 47 weeks) after the onset of long COVID. The average duration from infection to the onset of long COVID was 65 ± 42.9 days (approximately 9 weeks) (Supplementary Table S1). The mean age at the diagnosis of long COVID was 44.5 years, and patients presented with an average of six symptoms at the time of COVID-19 diagnosis. More than two-thirds of the study population were women, primarily affected during the second wave. Approximately one third of the participants (26%) were probable cases of SARS CoV-2 infection. Close to half of the participants had the long form of long COVID, and about 10% of all cases required hospitalization (Table 1).

**Table 1.** Socio-demographic characteristics of the study population.

		*n* = 206
Age at diagnosis of COVID-19 (in years)	Mean (SD)	44.5(11.1)
Number of symptoms during infection		6.1(2.3)
Gender	Frequency	%
Female	157	76
Male	49	24
**SARS CoV-2 virus infection wave**		
2	107	52
1	83	40
3	10	5
4	6	3
**Confirmation of COVID-19 infection**		
Yes	151	74
No	53	26
**Hospitalization during infection**		
No	183	90
Yes	21	10
**Clinical form of long COVID**		
Long	84	49
Persistent	78	45
Post-COVID or post-viral syndrome	11	6
**Obesity**		
No	178	86
Yes	28	14
**Anxiety**		
No	143	69
Yes	63	31
**Depression**		
No	162	79
Yes	44	21

SD, standard deviation.

### Description of clinical and biological variables

In order to characterize long COVID and identify similarities and dissimilarities within each cluster identified, demographic, clinical and biological variables were explored. The most prevalent symptoms of long COVID at inclusion were concentration disorders, memory disorders, fatigue, phasic disorders, and sleep disorders (Supplementary Table S2).

Among biological variables, three were excluded from the factorial analysis due to missing data: d-dimers, creatine kinase, and lactic dehydrogenase. All biological values fell within the normal ranges except for elevated C-reactive protein (CRP), LDL cholesterol, and total cholesterol. The median CRP was 22 [6-45] mg/L, the mean total cholesterol was 202.5 ± 40.6 mg/dL and the mean LDL cholesterol was 117.9 ± 35.9 mg/dL (Supplementary Table S3).

### Partition of individuals

The HCPC was then performed after determining the optimal number of principal components to be analysed. Ideally, 80% of all components should explain the percentage of inertia (variance).

**Figure 2.**
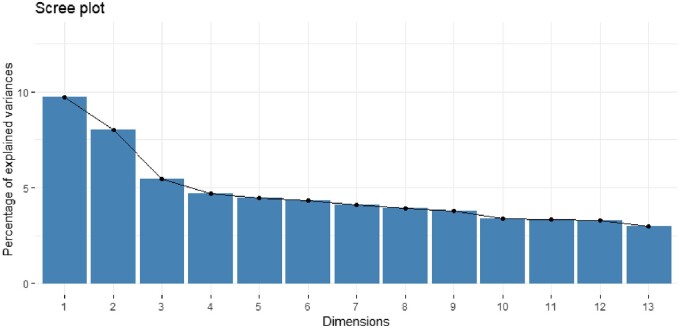
Optimal principal components for dimension reduction. y-axis: the eigenvalues of the factors or principal components. x-axis: the corresponding factor or component number.

While 21 dimensions were necessary to account for 80% of the explained variability, only 13 dimensions were retained because the other 8 dimensions had eigenvalues below 1%. The 13 dimensions are presented on a scree plot. The scree plot assists in identifying the optimal number of factors or components to retain for dimension reduction by examining the pattern of eigenvalues and the percentage of explained variance. Eigenvalues represent the amount of variance explained by each factor or component. The percentage of explained variance provides insights into how well a factor or principal component captures the overall variability in the data (Fig. 2).

The scree plot typically exhibits a steep drop in eigenvalues for the initial factors or components, followed by a relatively shallow, asymptotic “scree” or levelling off. This pattern helps in determining the point at which adding more factors or components provide diminishing returns in explaining additional variance.

### Identification of three clusters using HCPC

**Figure 3.**
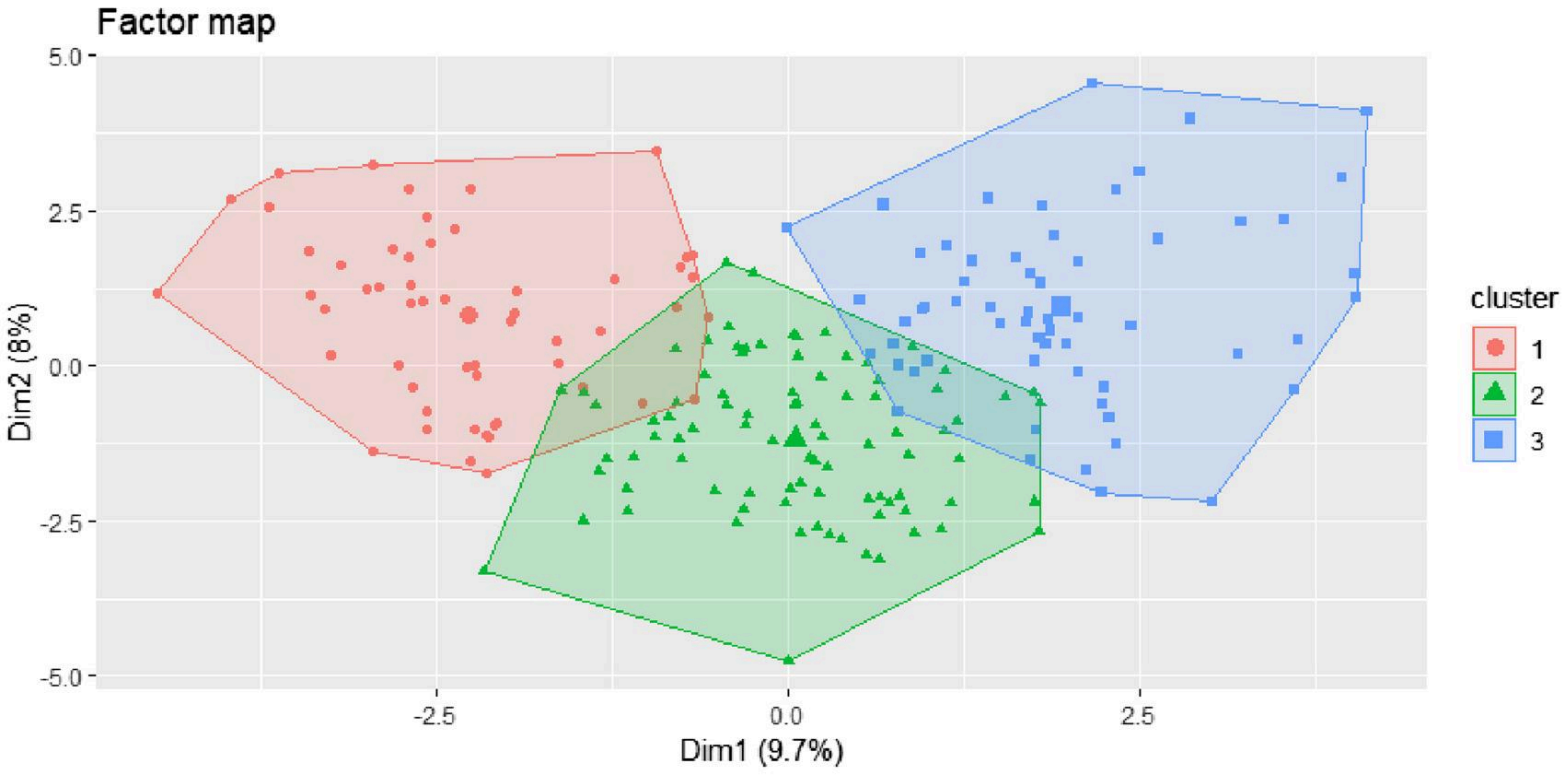
Factor map representing clusters of individuals.

The HCPC provides a representation of three clusters of individuals (Fig. 3).

Each point on the factor map represents an individual. Dim1 is the horizontal principal component with 9.7% of explained variance. Dim 2 is the vertical principal component with 8% of explained variance.

On average, 328 days after SARS-CoV-2 infection (11 months), the most distinctive symptoms of long COVID were partitioned into three clusters (Supplementary Table S4):

**Cluster 1 (Pain):** This cluster was characterized by the presence of myalgia. Predominantly male patients and persistent clinical phenotype were represented.
**Cluster 2 (Central Nervous System):** This cluster primarily featured pure neurocognitive disorders, including concentration, memory, and phasic disorders. It was more prevalent among females and aligned with the long clinical form.
**Cluster 3 (Anxiety/Depression, Pain, Peripheral Nervous System Disorders with Dysautonomia, Central Nervous System Disorders with Neurocognitive Disorders, Digestive System Disorders):** This cluster exhibited a diverse range of symptoms, including concentration and memory disorders, phasic disorders, headaches, fatigue, anxio-depressive syndrome with sleep and mood disorders, signs of dysautonomia (POTS, dyspnoea, exercise intolerance, palpitations), visual disorders, hearing disorders, taste disorders, paraesthesia, myalgias, joint and chest pain, digestive disorders (nausea, vomiting, abdominal pain), and dizziness.

## Discussion

This study aimed to categorize the various symptoms of long COVID to identify commonalities and differences between individuals, and to subsequently establish a possible connection with clinical forms. Our results show that two of the three clusters identified are linked to the two main clinical forms represented in the sample.

### General patterns of long COVID

Female sex, a history of psychiatric conditions, and allergies were identified as the primary antecedents of long COVID [3, 60]. Anxiety and depression antecedents were present in 31% and 21% of patients in this cohort, underscoring the interplay between physical and mental aspects in the burden of long COVID, which aligns with the biopsychosocial model [61].

This study's sample predominantly represented the second wave of COVID-19 infection in Belgium (between April 9, 2020, and February 15, 2021), followed by the first wave (before April 9, 2020). This may suggest that the wild-type strain of SARS-CoV-2 is responsible for long COVID, while the Alpha and Delta variants are primarily associated with the third and fourth waves [62]. However, as patient recruitment is ongoing, it remains possible that patients from subsequent waves will be included, and thus, it cannot be definitively stated that the first two waves are the primary contributors to long COVID.

The most common symptoms of long COVID in this cohort, on average 328 days after the onset of SARS-CoV-2 infection, included neurological disorders (especially concentration and memory issues), fatigue, and sleep disorders. These results are consistent with those reported in Germany, where these symptoms were identified six to twelve months after infection [63].

Furthermore, elevated levels of C-reactive protein were found in long COVID patients compared to those without long COVID who had experienced SARS-CoV-2 infection, based on a meta-analysis of over 20 inflammatory and vascular biomarkers [64]. These findings align with the results of this study, where C-reactive protein was elevated in some patients.

### Symptoms cooccurrence analysis

In terms of clustering, several studies assessing symptoms cooccurrence have been conducted, each with different patient profiles (hospitalized and/or non-hospitalized populations), with varying study periods, and different methodologies.

#### Two months after infection

Danesh *et al*. found two clusters. Cluster 1, the neuropsychiatric one had non-significant otorhinolaryngologic and Gastro intestinal symptoms, but significant neurologic and psychiatric symptoms which included: brain fog, confusion, concentration difficulties, headache, memory loss, paraesthesia, word finding difficulty, and other. Significant psychiatric symptoms included anxiety, depressed mood, insomnia and other. Other psychiatric symptoms included substance abuse, survivor guilt, bizarre dreams, and panic attacks. The pulmonary cluster included cough and dyspnoea with both clusters having non-significant cardiovascular symptoms [65].

#### Three months after infection

For in and outpatients, Wong *et al*. using latent class analysis found that Class 1 was characterized mainly by patients who had fatigue and dyspnoea, Class 2 by anxiety and depression, and Class 3 by fatigue, dyspnoea, anxiety, and depression [11].

Sahanic *et al*. found in outpatients approximately 3 months after infection 3 clusters: (i) hyposmia/anosmia phenotype, encompassing closely co-occurring smell and taste disorder; (ii) fatigue phenotype, including fatigue, tiredness, and memory and concentration deficits; and (3) MOP (Multiorgan phenotype) with pulmonary, gastrointestinal, neurocognitive, and cardiovascular disorders. [18]

Whitaker *et al.*, three months after infection, found 2 clusters in England: Cluster L1 (*n* = 15 799) had persistent tiredness which co-occurred with muscle aches, difficulty sleeping, and shortness of breath. Cluster L2 (*n* = 4441) had high prevalence of respiratory symptoms including shortness of breath and tight chest, as well as chest pain. [66]

Gottlieb *et al.* in the USA, both in- and outpatients, reported four phenotypes (at 3 and 6 months follow-up): minimal symptoms phenotype, tired/headache/musculoskeletal (MSK) phenotype, loss of smell/taste phenotype, and many symptoms across multiple systems phenotype [19].

Nayani *et al*. in Belgium, 3 months after infection, found three phenotypes in both in- and outpatients: The first class gathered 19% of the participants reporting PCC symptoms. Participants in this class had the highest probability of having a loss of smell and taste (81% and 51%, respectively) and were therefore labelled class 1 as ‘loss of smell and taste’. The second class was the largest and gathered 67% of the participants with PCC. The symptoms with the highest probabilities in this class were headache (51%) and memory problems (46%). These probabilities were higher than in classes 1 and 3. Fatigue-exhaustion was also common in this class (46%). They labelled this second class ‘neurological symptoms’. The third and last class was the smallest and gathered 14% of the participants with PCC. Participants in this class had a high probability of having a large number of symptoms (median = 4/IQR = 3–7, in comparison with the first class: median = 2/IQR = 1–4 and second class: median = 3/IQR = 2–5). The symptoms with the highest probabilities in this class were fatigue-exhaustion (89%), muscle pain (62%), dyspnoea (54%), sleeping problems (51%), headache (48%), joint pain (42%), memory problems (41%), dizziness (38%), constipation (31%), and palpitations (31%). They have chosen to label this third class ‘multiple symptoms’ [14].

In Spain, Torrell *et al*., three months and more after infection, found five phenotypes in outpatients: multisystemic (34.59%), multisystemic—predominantly dysautonomous (17.79%), heterogeneous (23.98%), taste and smell (5.97%), and menstrual and sexual alterations (17.68%). Heterogeneous is a cluster in which no single system is predominantly affected [24].

#### Four months after infection

Peluso *et al*. in USA found two phenotypes in both in and outpatients: participants in Cluster 1 have higher prevalence of all symptom domains except for respiratory, indicating that symptom presence across the seven domains is, in general, positively correlated. Cluster 1 is particularly distinguished from Cluster 2 in its greater absolute difference in prevalence of fatigue, cardiopulmonary symptoms, and gastrointestinal symptoms [67].

In Spain, Mateu et al found in and outpatients 3.7 months after infection 3 phenotypes: individuals in cluster A (40.8% of subjects) presented primarily with fatigue; those in cluster B (44.6% of subjects) had fatigue plus dyspnoea, neurocognitive complaints, headache, myalgia, arthralgia, chest pain and tachycardia; individuals in cluster C (14.2% of subjects) had the same dominant symptoms of cluster B plus skin and smell alterations, dysphagia, diarrhea, and neurosensitive symptoms. [68]

#### Six months after infection

Ballouz *et al*. in Switzerland reported four clusters in both in and outpatients: diverse systemic (*n* = 219 of participants with post-COVID conditions), neurocognitive (*n* = 47), cardiorespiratory (*n* = 23), and MSK symptoms (*n* = 19) [69].

Kenny *et al*. in Ireland identified individuals with symptoms persisting >4 weeks from acute COVID-19 at 24 and 18 weeks after infection and they were divided into two groups based on timing of acute infection: pre-Alpha VOC (Variants of Concern), denoted wild type (WT) group and post-Alpha VOC (incorporating alpha and delta dominant periods) denoted VOC group. In both groups, multilatent class analysis identified three similar clusters; a MSK cluster characterized by joint pain and myalgia, a cardiorespiratory cluster, and a less symptomatic cluster. Differences in characteristic symptoms were only seen in the cardiorespiratory cluster where a decrease in the frequency of palpitations (10% vs 34% *P* = .008) and an increase in cough (63% vs 17% *P* < .001) in the VOC compared to WT groups was observed [15, 70].

Approximately 7.2 months after infection, Ziauddeen *et al*. found two phenotypes in a multicentred study including outpatients exclusively: ongoing symptom cluster (OSC) 1 predominantly including participants with cardiopulmonary symptoms (shortness of breath, chest pain, chest pressure/tightness, palpitations), neuro-cognitive symptoms (brain fog, poor concentration, memory problems, dizziness), and exhaustion (*n* = 2243, 88.8%); and OSC2, a minority cluster, including multisystem ongoing symptoms (*n* = 283, 11.2%). The most common ongoing symptoms in OSC2 include shortness of breath, chest pain, chest pressure/tightness, palpitations (cardiopulmonary); brain fog, poor concentration, memory problems, dizziness, confusion, pins and needles (neuro-cognitive); sore throat, hoarse voice, nasal symptoms (nose/throat); headache, joint pain, leg pain, muscle ache (pain); and exhaustion, sleep disturbance and chills (systemic) [71].

#### Eleven months after infection

Silverberg *et al*. found four phenotypes in outpatients using latent class analysis: Class-4 had the highest membership probability (82.6% of the survey cohort) and consisted of very low probabilities of any chronic COVID-19 symptoms. Class-2 had the next highest membership probability (9.2% of the survey cohort) and had higher probabilities of chronic fatigue, muscle aches, weakness, shortness of breath, cough, headaches, rapid heart rate, change in hearing, and stomach pain. Class-3 (4.6% of the survey cohort) had higher probabilities of chronic fatigue, change in smell and taste, muscle aches, weakness, nausea, and dizziness. Class-1 (3.6% of the survey cohort) had higher probabilities of chronic changes of smell and taste [72].

#### Twelve months after infection

Frontera *et al*. found three clusters in inpatients: Cluster 1 (*N* = 38, 31%) included patients with few symptoms, most often headache and anxiety though at lower rates than reported in other clusters; Symptom Cluster 2 (*N* = 16, 13%) consisted of patients with many symptoms, including high levels of anxiety and depression; and Symptom Cluster 3 (*N* = 68, 56%) included patients who predominantly reported shortness of breath, headache, and cognitive abnormalities. Patients in Symptom Cluster 2 had the highest number of symptoms and stressors, were least likely to report that their symptoms did not affect routine activities and had worse 12-month quantitative outcomes as compared to the other groups [16].

Fischer *et al*. in Luxembourg found four phenotypes in both in and outpatients: a first cluster constituted most of neurological symptoms, a second cluster was constituted by pain-related and cardiovascular symptoms (muscle or joint pain in the upper limbs was frequently reported together with muscle or joint pain in the lower limbs, back pain, feeling of tightness in the chest, or arrhythmia), a gastro-intestinal cluster and finally a loss of smell and taste cluster [73].

Small sample size, distinct waves studied, transversal versus longitudinal analysis, period post infection of the study can yield different results especially with inflammatory biomarkers such as CRP [74].

Moreover, due to the dynamic nature of clusters (evolving phenotypes with time) [18, 19, 24], we will discuss only few studies, from 6 to 12 months after infection. The clusters which are present are neurocognitive, cardiorespiratory, MSK (or pain cluster), multisystemic (including anxiety and depression, gastrointestinal symptoms), and the inconsistent “smell and taste” cluster.

This study didn’t yield a cardiorespiratory cluster due to the fact that symptoms such as chest pain, chest tightness, palpitations were considered as consequences of dysautonomia in absence of organic lesions (investigated by cardiologists).

The Anosmia/agueusia cluster, which is inconsistent, appears usually early and disappear within 3 months [75], or it appears later associated to systemic symptoms, thus suggesting movements across clusters [24, 72]. Anosmia/agueusia tends to be predominant in patients with polymerase chain reaction (PCR) positive tests [24].

We did not exclude patients with probable SARS CoV-2 infection because one study classified the patients into two groups, ELISPOT high and low, based on ELISPOT S1, S2, and N values, and found that the number of persistent symptoms was significantly higher in the ELISPOT-low group than those in the ELISPOT-high group (*P* = .0497). These results suggest that patients with COVID-19 having insufficient T-cell immunity to SARS-CoV-2 after recovery (probable cases of SARS CoV-2 infection) are more likely to have persistent COVID-19 symptoms [76].

Several studies also included and compared patients without positive PCR tests. Davis *et al*. in a multicentred study analysed in and outpatients with probable and confirmed cases of SARS CoV-2 infection and found that except for loss of smell and taste, the prevalence and trajectory of all symptoms were similar between groups with confirmed and suspected COVID-19 [22].

Another study also analysed symptoms by microbiological diagnostic testing and found no significant differences in symptoms between participants who had a positive Rapid Antigenic Test or PCR and those who did not, except for olfactory alterations that were more common during the first 21 days in those who had a positive test (63.6%) than in those who did not (50.8%), taste and smell alterations (53.9% of those who had a positive test and 39.3% of those wo had not) [24].

However, Ziauddeen *et al*. found in a transversal study including outpatients significant differences in reported prevalence of ongoing symptoms in those with and without lab-confirmation that include altered/loss of sense of smell or taste and brain fog which were higher in those with lab-confirmation than without, whereas abdominal pain, nausea, chest pain, chest tightness, chills, hoarse voice, sore throat, sneezing, and pins and needles were lower in those with lab-confirmation than without [71]. These differences need further studies to be explained.

### Pathophysiological mechanisms underlying clusters

The COVID-19 pandemic has significantly heightened concerns about long-term impacts on cognitive function and mental health [76–79]. Neurocognitive and psychiatric symptoms appear strongly correlated with persistent systemic inflammation in long COVID [80]. Furthermore, research by Sahanic *et al*. revealed a link between anxiety, depression, persistent symptoms, and slower recovery rates [81], while another by Vartanian *et al*. revealed that clusters of people with ongoing multiple mental health challenges, cognitive struggles (36% of sample), and fewer physical symptoms were still significantly impacted on their social lives and day-to-day functioning 5–6 months after infection [82]. Kim *et al*. found that neuropsychiatric symptoms tend to persist over time, up to 2 years after infection [83]. These findings underscore the critical need to understand and mitigate this persistent inflammation to alleviate the duration and severity of these debilitating symptoms.

The period from initial COVID-19 infection to the development of long COVID presents a critical window for intervention. Several studies highlight this acute phase as crucial in the pathogenesis of long COVID [81, 84–86]. For instance, one study observed a persistent inflammatory state in a subset of patients present during both the acute and convalescent phases (7 weeks), associated with neurological symptoms like fatigue, muscle weakness, and difficulty swallowing [85] while another study highlighted the importance of T cell immunity for symptom resolution, suggesting that its measurement after recovery could predict long-term outcomes [86].

Research indicates that the severity of the COVID-19 infection, hospitalization, and the time since the initial symptoms are associated with the prevalence of cognitive complaints and measurable cognitive deficits. However, the precise mechanisms underlying these impairments remain unclear. Potential contributors may include inflammation, neuroinflammation, cytokine storms, and the effects of critical care and sedation [77].

Cluster 2, characterized by pure neurocognitive disorders (concentration, memory, and phasic disorders), might be a consequence of cerebral hypoxia, as this has been suggested as a pathophysiological mechanism for neurological symptoms associated with long COVID [87, 88]. In this group, an associated inflammatory component may also play a role in the onset of neurocognitive disorders. In this context, the interferon (IFN)-complement system has been proposed as the inflammatory mechanism underlying age-related neurocognitive disorders [89].

Cluster 3 (anxio-depressive syndrome, fatigue, mood and sleep disorders, pain, neurocognitive disorders, dysautonomia, peripheral nervous system disorders, and digestive disorders) shares similar symptoms with ME/CFS (Myalgia encephalomyelitis/chronic fatigue syndrome). One study even suggests that long COVID patients who express symptoms like ME/CFS are prone to anxiety and depression due to sarcosine and serine reduction [90]. This cluster of patients may be also the result of a combination of factors, including viral persistence in olfactory bulb or other peripheral nerves (neuroinvasiveness and neurotropism [91]) followed by neuroinflammation as 80% of olfactive and gustative disorders usually disappear within 3 months [75].

Cluster 1, characterized by myalgia-type pain, could be likened to fibromyalgia syndrome. Some authors have even described long COVID as fibromyalgia [92]. Proposed mechanisms for fibromyalgia include neuroinflammation and dysautonomia, although dysautonomia was not sufficiently present in this cluster to characterize it.

To gain a better understanding of the pathophysiological mechanisms within these clusters, it would have been necessary to measure and characterize various unconventional biomarkers to explore different pathways:

- IL-6, IFN, and complement, which can help assess neuroinflammation [64, 89],
- Von Willebrand factor, ADAMTS-13 protein (a disintegrin and metalloproteinase with thrombospondin type 1 motifs, member 13) as biomarkers of endothelial dysfunction [60, 93],
- HUTT (Head-up tilt table) test to explore orthostatic intolerance, providing a more precise evaluation of dysautonomia [94].

## Study limitations

This study has encountered biases, as it was conducted retrospectively. The data were collected from patient assessments and sometimes did not include all the necessary information. Ideally, a standardized assessment in both COVID and post-COVID situations would have been more accurate and reduced potential information biases. Additionally, to reduce selection biases or for a more representative sample, it would have been necessary to include all clinical forms of long COVID, including hospitalized patients with severe forms of COVID-19, who represent a significant proportion of individuals affected by long COVID. Although studies have noticed few or no differences in long COVID symptoms between suspected and confirmed cases of COVID-19, it would have been appropriate to conduct analysis separately between these two groups in this study.

We were limited by the lack of sufficient sociodemographic, socioeconomic, and psychological relevant factors which could help exploring determinants that can influence the course of long COVID as the biopsychosocial model is believed to be involved in the pathophysiology of this condition. The fact that we included patients with probable SARS CoV 2 infection could also lead to potential bias. Higher prevalence of anxiety and depression in antecedents in this sample could also introduce bias. Omitted variables such as d-dimer and vitamin D in multidimensional analysis could have also led to bias in characterization of biological variables.

## Conclusion

This research yielded two main findings: the identification of three clusters of long COVID patients, aligning with two principal clinical forms. Usual biological laboratory tests could not differentiate between these clusters. Therefore, clinicians should conduct meticulous clinical examinations and rule out other diseases to identify different clinical forms of long COVID. Further studies will be necessary to understand the pathophysiological mechanisms or genetic differences present in each cluster/clinical form, and to assess long COVID between probable and confirmed cases of SARS CoV-2 infection. Moreover, a consensual definition and classification could alleviate the diagnosis of this condition.

## Supplementary Material

bpae076_Supplementary_Data

## Data Availability

The data presented in this study are available on request from the corresponding author.
