# Supplementary material for: Cluster analysis identifies long COVID subtypes in Belgian patients
Source: Biol Methods Protoc. 2024 Oct 9;9(1):bpae076. doi: 10.1093/biomethods/bpae076 (PMC11522879; doi:10.1093/biomethods/bpae076)
Supplement: bpae076_Supplementary_Data [file bpae076_supplementary_data.zip › S4_Table.docx]

S4 Table. **Clusters repartition with v-tests**

|  | **v-tests** | | |
| --- | --- | --- | --- |
| **Modalities** | **Cluster 1**  **n=49** | **Cluster 2**  **n=81** | **Cluster 3**  **n=75** |
| Type=persistent | 3.73 | -2.97 | -0.44 |
| Myalgia=yes | 2.39 | -6.28 | 3.96 |
| Gender=male | 2.6 | -2.82 | 0.41 |
| Gender=female | -2.6 | 2.82 | -0.41 |
| Type=long | -3.19 | 2.23 | 0.69 |
| HDL cholesterol | -4.42 | 5.05 | -1.17 |
| Phasic disturbances=yes | -8.19 | 2.36 | 5.45 |
| Memory disturbances=yes | -9.24 | 4.47 | 5.13 |
| Concentration disturbances=yes | -10.5 | 6.17 | 4.58 |
| Vertigo=yes | -2.25 | -1.81 | 3.86 |
| Digestive troubles=yes | 0.95 | -3.97 | 3.13 |
| Anxio-depressive syndrome=yes | -0.961 | -3.62 | 5.29 |
| Mood disturbances=yes | -3.48 | -1.12 | 4.4 |
| Sleep disturbances=yes | -5.42 | 0.04 | 5.19 |
| CRP | 1.69 | -4.33 | 3.09 |
| LDL cholesterol | 0.521 | -2.62 | 2.37 |
| Chest pain=yes | 1.17 | -3.31 | 2.28 |
| Joint pain=yes | 0.35 | -5.47 | 5.15 |
| Dyspnoea=yes | -0.05 | -3.47 | 3.84 |
| Palpitations=yes | -2.65 | -3.17 | 5.57 |
| Exercice intolerance=yes | -2.68 | -1.17 | 3.58 |
| Dysautonomia=yes | -0.72 | -4.27 | 5.16 |
| Paresthesia=yes | -1.21 | -2.33 | 3.59 |
| Visual disturbances=yes | -2.05 | -2.2 | 4.14 |
| Taste disturbances=yes | 0.92 | -3.1 | 2.25 |
| Hearing disturbances=yes | -3.08 | -0.82 | 3.5 |

In the context of the HCPC (Hierarchical Clustering on Principal Components) algorithm implemented in the FactoMineR package in R, it is not based on traditional hypothesis testing like p-values. In HCPC, the clusters are determined based on distances between observations in a high-dimensional space, typically after dimensionality reduction techniques like Factor Analysis of Mixed Data (FAMD). The v-test value represents the contribution of variables to the formation of clusters. A higher v-test value, usually > 1.96, indicates that the variable is more relevant in discriminating between clusters. The significance of the v-test values in HCPC depends on the context of the data and the specific goals of the analysis. The exact interpretation of high v-test values as indicating variables that strongly differentiate between clusters would depend on the understanding of the data and the goals of the analysis.
