# Supplementary material for: Cluster analysis identifies long COVID subtypes in Belgian patients
Source: Biol Methods Protoc. 2024 Oct 9;9(1):bpae076. doi: 10.1093/biomethods/bpae076 (PMC11522879; doi:10.1093/biomethods/bpae076)
Supplement: bpae076_Supplementary_Data [file bpae076_supplementary_data.zip › S1_Table.docx]

S1 Table. **Time variables**.

| Variables (Days) | N= 206¹ |
| --- | --- |
| Time from onset of infection to completion of check-ups | 126.6 (112.4) |
| Time from onset of Long Covid to inclusion in the study | 290.4 (178.3) |
| Time from onset of infection to beginning of Long Covid | 65.7(43.0) |
| Time from infection to inclusion in the study | 328.0 (176.0) |

¹ Mean (SD). SD= Standard Deviation.
