# Supplementary material for: Cluster analysis identifies long COVID subtypes in Belgian patients
Source: Biol Methods Protoc. 2024 Oct 9;9(1):bpae076. doi: 10.1093/biomethods/bpae076 (PMC11522879; doi:10.1093/biomethods/bpae076)
Supplement: bpae076_Supplementary_Data [file bpae076_supplementary_data.zip › S3_Table.docx]

S3 Table. **Biological variables**

| **Variables** | **Biological Values** |
| --- | --- |
|  | N= 206¹ |
| C-reactive protein (mg/L) | 22.0 [6.0 - 46.5] |
| Creatine kinase (IU/L) | 67.0 [50.0 - 89.0] |
| Alkaline phosphatase (IU/L) | 63.0 [54.0 - 74.0] |
| Lactate dehydrogenase (IU/L) | 177.0 [156.5 - 210.0] |
| Aspartate aminotransferase (U/L) | 19.0 [16.0 - 23.0] |
| Alanine aminotransferase (U/L) | 13.0 [7.0 - 25.0] |
| D-dimers (µg/L) | 301.0 [216.0 - 409.0] |
| Total cholesterol (mg/dL) | 202.5 (40.6) |
| HDL cholesterol (mg/dL) | 60.1 (16.2) |
| LDL cholesterol (mg/dL) | 117.9 (35.9) |
| Lymphocytes (10³/µL) | 2.2 (1.2) |
| Neutrophils (10³/µL) | 3.9 (1.8) |
| Platelets (10³/mm³) | 263.6 (66.4) |
| Ferritin (µg/L) | 127.8 (113.3) |
| Blood glucose (mg/dL) | 93.0 (20.3) |
| Creatinine (mg/dL) | 0.8 (0.5) |
| Calcium (mmol/L) | 2.5 (1.0) |
| Vitamin D (ng/mL) | 32.2 (16.3) |

¹Median [IQR]; Mean (SD). IQR= Interquartile Range; SD= Standard Deviation; mg= milligrams; L=litre; IU=U= International Units; µg= micrograms; mg= milligrams; dL= decilitre; HDL= High Density Lipoprotein; LDL= Low Density Lipoprotein; µL= microlitre; mm= millimetres; mmol= millimoles; ng= nanograms.
