# Supplementary material for: Cluster analysis identifies long COVID subtypes in Belgian patients
Source: Biol Methods Protoc. 2024 Oct 9;9(1):bpae076. doi: 10.1093/biomethods/bpae076 (PMC11522879; doi:10.1093/biomethods/bpae076)
Supplement: bpae076_Supplementary_Data [file bpae076_supplementary_data.zip › S2_Table.docx]

S2 Table. **Long COVID symptoms at inclusion**

| **Variables** | **Long Covid symptoms at inclusion**  **N=205¹** |
| --- | --- |
| **Concentration troubles** |  |
| Yes | 74 |
| No | 26 |
| **Memory disturbances** |  |
| Yes | 73 |
| No | 27 |
| **Phasic disturbances** |  |
| Yes | 55 |
| No | 45 |
| **Sleep disturbances** |  |
| No | 55 |
| Yes | 45 |
| **Mood disturbances** |  |
| No | 73 |
| Yes | 27 |
| **Taste disturbances** |  |
| No | 89 |
| Yes | 11 |
| **Olfactory disturbances** |  |
| No | 90 |
| Yes | 10 |
| **Vision disturbances** |  |
| No | 85 |
| Yes | 15 |
| **Hearing disturbances** |  |
| No | 88 |
| Yes | 12 |
| **Fatigue** |  |
| Yes | 71 |
| No | 29 |
| **Exercice intolerance** |  |
| No | 86 |
| Yes | 14 |
| **Alcohol intolerance** |  |
| No | 98 |
| Yes | 2 |
| **Headaches** |  |
| No | 71 |
| Yes | 29 |
| **Paresthesia** |  |
| No | 80 |
| Yes | 20 |
| **Dizziness** |  |
| No | 88 |
| Yes | 12 |
| **Hair and eyelash loss** |  |
| No | 94 |
| Yes | 6 |
| **Dysautonomia** |  |
| No | 72 |
| Yes | 28 |
| **Chest pain** |  |
| No | 84 |
| Yes | 16 |
| **Joint pain** |  |
| No | 77 |
| Yes | 23 |

¹Frequency (%)
